# Deregulated miRNAs Contribute to Silencing of B-Cell Specific Transcription Factors and Activation of NF-κB in Classical Hodgkin Lymphoma

**DOI:** 10.3390/cancers13133131

**Published:** 2021-06-23

**Authors:** Julia Paczkowska, Joanna Janiszewska, Adam Ustaszewski, Julia Bein, Marcin Skalski, Agnieszka Dzikiewicz-Krawczyk, Natalia Rozwadowska, Martin-Leo Hansmann, Sylvia Hartmann, Maciej Giefing

**Affiliations:** 1 Institute of Human Genetics, Polish Academy of Sciences, 60-479 Poznan, Poland; julia.paczkowska@igcz.poznan.pl (J.P.); joanna.janiszewska@igcz.poznan.pl (J.J.); adam.ustaszewski@igcz.poznan.pl (A.U.); marcinskalski1993@gmail.com (M.S.); agnieszka.dzikiewicz-krawczyk@igcz.poznan.pl (A.D.-K.); natalia.rozwadowska@igcz.poznan.pl (N.R.); 2 Dr. Senckenberg Institute of Pathology, Goethe University Frankfurt, D-60590 Frankfurt, Germany; julia.bein@kgu.de (J.B.); martin-leo.hansmann@kgu.de (M.-L.H.); s.hartmann@em.uni-frankfurt.de (S.H.)

**Keywords:** classical Hodgkin lymphoma, microdissection, miRNA, loss of B-cell phenotype, NF-κB, B-cell transcription factors

## Abstract

**Simple Summary:**

The role of transcriptionally deregulated miRNAs (microRNAs) in classical Hodgkin lymphoma (cHL) is still not fully understood. To address this issue, we have performed global miRNA expression profiling of commonly used cHL cell lines and we present a complete cHL miRNome (microRNome). Within this group, we identify miRNAs recurrently deregulated in cHL cell lines, and compare them to non-Hodgkin lymphoma cell lines and sorted normal CD77^+^ germinal centre B-cells. Moreover, we show that several of the recurrently overexpressed miRNAs in cHL cell lines, and also primary microdissected HRS (Hodgkin and Reed-Sternberg) cells, target known B-cell-related transcription factors and NF-κB inhibitors. These findings provide evidence that deregulated miRNAs contribute to the loss of B-cell phenotype and NF-κB activation observed in this lymphoma.

**Abstract:**

A hallmark of classical Hodgkin lymphoma (cHL) is the attenuation of B-cell transcription factors leading to global transcriptional reprogramming. The role of miRNAs (microRNAs) involved in this process is poorly studied. Therefore, we performed global miRNA expression profiling using RNA-seq on commonly used cHL cell lines, non-Hodgkin lymphoma cell lines and sorted normal CD77^+^ germinal centre B-cells as controls and characterized the cHL miRNome (microRNome). Among the 298 miRNAs expressed in cHL, 56 were significantly overexpressed and 23 downregulated (*p* < 0.05) compared to the controls. Moreover, we identified five miRNAs (hsa-miR-9-5p, hsa-miR-24-3p, hsa-miR-196a-5p, hsa-miR-21-5p, hsa-miR-155-5p) as especially important in the pathogenesis of this lymphoma. Target genes of the overexpressed miRNAs in cHL were significantly enriched (*p* < 0.05) in gene ontologies related to transcription factor activity. Therefore, we further focused on selected interactions with the *SPI1* and *ELF1* transcription factors attenuated in cHL and the NF-ĸB inhibitor *TNFAIP3*. We confirmed the interactions between hsa-miR-27a-5p:SPI1, hsa-miR-330-3p:ELF-1, hsa-miR-450b-5p:ELF-1 and hsa-miR-23a-3p:TNFAIP3, which suggest that overexpression of these miRNAs contributes to silencing of the respective genes. Moreover, by analyzing microdissected HRS cells, we demonstrated that these miRNAs are also overexpressed in primary tumor cells. Therefore, these miRNAs play a role in silencing the B-cell phenotype in cHL.

## 1. Introduction

Global epigenetic reprograming, mediated by alterations in DNA methylation, distinguishes classical Hodgkin lymphoma (cHL) from not only normal germinal centre B (GCB) cells, but also from other germinal centre derived B-cell lymphomas [1]. Aberrant DNA methylation is crucial for the survival of the neoplastic cells of cHL—the Hodgkin and Reed–Sternberg (HRS) cells—as shown by the regression of a relapsed metastatic cHL in a patient treated with the demethylating agent 5-azacytidine for myelodysplastic syndrome, or by the enhanced efficacy of immune checkpoint inhibitors after therapy using demethylating agents [2,3]. The canonical mechanism through which epigenetic alterations contribute to neoplastic disease is the hypermethylation of the promoter regions of tumor suppressor genes (TSGs), leading to their transcriptional silencing or the activation of oncogenes by loss of DNA methylation. However, in cHL, in addition to the usual hypermethylation of TSG promoters, DNA hypermethylation was reported to contribute to the loss-of-B-cell phenotype of HRS cells by silencing the B-cell specific transcription factors [4,5]. In this context, most studies focus exclusively on DNA methylation; in contrast, the role of the other epigenetic mechanism, namely miRNA in the regulation of B-cell specific transcription factors in the pathogenesis of cHL has remained largely understudied. However, the importance of miRNAs in normal B-cell development shown in many studies allows us to speculate on the putative role of miRNA deregulation in silencing B-cell specific transcription factors and the consequent loss-of-B-cell identity of HRS cells [6,7,8,9].

Therefore, in this study, we performed miRNA expression profiling using small RNA sequencing (RNA-seq) experiments in cHL cell lines, sorted GCB cells and non-Hodgkin lymphoma (NHL) cell lines. These data were used to (i) establish a complete cHL-specific miRNA expression profile and (ii) identify the group of miRNAs deregulated in cHL. Moreover, using in silico enrichment analysis and experimental validation, we identified several deregulated miRNAs involved in the attenuation of B-cell specific transcription factors and the constitutive activity of NF-κB in this lymphoma. Our study suggests that, in addition to DNA methylation, deregulation of miRNAs is another epigenetic process leading to the loss B-cell phenotype.

## 2. Materials and Methods

### 2.1. Lymphoma Cell Lines, GCB Cells and Primary cHL Biopsies

Three cHL cell lines (U-HO1, SUP-HD1, HDLM-2), 10 NHL cell lines (Burkitt lymphoma: Raji, Ca46, Daudi, Namalwa, Ramos; Diffuse Large Cell Lymphoma: OCI-LY1, OCI-LY3, OCI-LY7, SU-DHL-6; B Cell Lymphoma: Val) were obtained from the DSMZ resource center (Braunschweig, Germany). Moreover, four cHL cell lines (L-428, L-540, L-1236, KM-H2) obtained from collaborating partners were STR-typed to confirm their identity. The GCB6-16 cell line derived from immortalized GCB cells was a kind gift from Prof. Ralf Küppers (University of Duisburg-Essen, Essen, Germany) and Prof. Martin-Leo Hansmann (University of Frankfurt/Main, Frankfurt/Main, Germany). Cell lines were grown in RPMI medium supplemented with 10% FBS (U-HO1, SUP-HD1, L-428, L-1236, KM-H2, RAJI, CA-46, DAUDI, NAMALWA, VAL) or 20% FBS (L-540, HDLM-2, RAMOS, SU-DHL-6, OCI-LY3, GCB6-16). In contrast, the OCI-LY1 and OCI-LY7 cell lines were grown in IMDM medium (Thermo Fisher Scientific, Waltham, MA, USA) supplemented with 20% FBS.

Normal CD77^+^ GCB cells were purified from fresh tonsils obtained from tonsillectomies of chronic hyperplastic tonsillitis using magnetic activated cell sorting (MACS; Miltenyi Biotech, Bergisch Gladbach, Germany), as reported previously [10]. Primary HRS cells and adjacent bystander cells used as controls were microdissected from frozen lymph node sections HE (hematoxylin and eosin) stained of cHL patients using the PALM Robot MicroBeam laser microdissection system (PALM, Bernried, Germany) and pooled into groups of 1000 cells. The study was approved by the local ethics committee of the Goethe University Hospital (157/17 from 6 April 2017).

### 2.2. NGS Based miRNA Profiling

Total RNAs from cell lines were isolated using the Trizol reagent (Thermo Fisher Scientific, Waltham, MA, USA) based on the Chomczyński method [11]. RNAs from sorted GCB cells and microdissected HRS cells were isolated using miRNeasy Mini Kit (Qiagen, Hilden, Germany). RNA quality and quantity was measured using the NanoDrop ND100 (Thermo Fisher Scientific, Waltham, MA, USA) and analyzed using the Agilent Bioanalyzer (Agilent, Santa Clara, CA, USA).

Total RNAs were shipped to the BGI company (Shenzhen, China) for small RNA sequencing. The NEBNext^®^ Small RNA Library Prep Set for Illumina^®^ (New England Biolabs Ipswich, MA, USA) was used for library preparation and small RNA sequencing experiments were performed using the HiSeq 4000 platform (Illumina, San Diego, CA, USA) and the SE50 (single-end 50 bp) sequencing protocol. Quality control was performed using FastQC software (Andrews S. (2010). FastQC: a quality control tool for high throughput sequence data. Available online at: http://www.bioinformatics.babraham.ac.uk/projects/fastqc, accessed on 14 May 2021) showing very high quality of the reads (Phred quality score above Q30). The obtained reads have been mapped to reference genome (GRCh37) using Bowtie (http://bowtie-bio.sourceforge.net/index.shtml, accessed on 31 March 2016) software, and the miRNA quality has been estimated with the CAP-miRSeq (http://bioinformatics.mayo.edu/research/cap-mirseq/, accessed on 31 March 2016) package. Further processing of the data including filtration, normalization and statistical calculations was performed using the edgeR package (Bioconductor) (PMID: **19910308**).

Two NGS experiments were performed. The first included cHL (*n* = 7) and NHL cell lines (*n* = 10) and the second included cHL (*n* = 3) and GCB samples (*n* = 10). We used counts per million (CPM) as a normalized determinant of miRNA expression. The CPM values of ≥10 in at least 3/7 cHL cell lines were regarded as indicative for the expression of a particular miRNA. Therefore, the miRNAome of cHL includes all detected miRNAs in the seven cHL cell lines fulfilling this criterion.

To identify miRNAs upregulated in cHL, we selected miRNAs (log FC > 1.5; *p* < 0.05) separately between (i) cHL and NHL and between (ii) cHL and GCB (differential expression analysis was performed using edgeR (PMID: **19910308**)). Only miRNA expressed at least in 3/7 cHL were included. Similarly, for the miRNAs downregulated in cHL, we selected miRNAs (log FC < −1.5; *p* < 0.05) separately between (i) cHL and NHL and between (ii) cHL and GCB. Only miRNAs expressed at least in 5/10 NHL and 5/10 GCB were included. With this filtering method, we received two sets of differently expressed miRNAs (cHL vs. NHL) and (cHL vs. GCB). By merging these two sets of miRNAs deregulated in cHL, we created a common set of deregulated miRNAs in cHL.

### 2.3. Real-Time qPCR Based miRNA Expression Analysis

The cDNA templates for real-time qPCR analyzes were synthesized from 10 ng of total RNA using the TaqMan^TM^ Advanced miRNA cDNA Synthesis Kit (Thermo Fisher Scientific, Waltham, MA, USA) according to supplier’s protocol. In detail, poly(A) tailing was added to miRNAs followed by adapter ligation and the universal reverse transcription. Lastly, cDNA was amplified with universal forward and reverse primers.

Real-time qPCR reactions were performed in triplicate using the Bio-Rad CFX96 Real-Time PCR System (Bio-RAD, Hercules, CA, USA) with TaqMan™ Fast Advanced Master Mix (Thermo Fisher Scientific, Waltham, MA, USA ) and the TaqMan™ Advanced miRNA Assays (Thermo Fisher Scientific, Waltham, MA, USA) according to the protocol provided by the manufacturer (Thermo Fisher Scientific, Waltham, MA, USA). Using the BioRad Genex software (Bio-RAD, Hercules, CA, USA), the expression of particular miRNAs was calculated in relation to the miR-191-5p and miR-361-5 reference miRNAs, or in relation to the miR-let-7g and miR-361-5p reference miRNAs in the case of the real-time qPCR performed in microdissected HRS cells (Table S1). The chosen reference miRNAs showed stable expression across analyzed cell lines based on the small RNA-seq data.

### 2.4. Identification of Putative Target Genes of the cHL Deregulated miRNAs

We used the Targetscan (http://www.targetscan.org, accessed on 31 July 2017) prediction tool to identify putative target genes to be regulated (miRNA-mRNA interaction) by the two groups of miRNAs, the overexpressed and the downregulated in cHL. Target mRNA genes harboring a respective 8-mer and/or 7mer-m8 miRNA binding site in their 3′UTR regions with a weighted context score below −0.5 were selected in each group. The two groups of target genes were analyzed for enrichments in biological process (gene ontology (GO) analysis) using the PANTHER database (http://pantherdb.org/, accessed on 31 July 2017), the STRING database (http://string-db.org, accessed on 31 July 2017) and the DAVID database (https://david.ncifcrf.gov, accessed on 14 May 2021).

### 2.5. Validation of miRNA Target Genes

#### 2.5.1. Vector Preparation

Fragments of the 3′UTR regions of selected genes (*ELF1, SPI1, TNFAIP3*), containing binding sites for the analyzed miRNAs were synthesized (Genomed, Warsaw, Poland) and cloned into the pmirGLO Dual-Luciferase miRNA Target Expression Vector (Promega, Madison, WI, USA) followed by JM109 competent cells transformation (Promega, Madison, WI, USA). Wild type (WT) or mutant (MUT) variants of miRNA binding sites located in the 3′UTRs were designed, as proposed by Mets et al. [12]. WT constructs contained miRNA binding sites of the respective 3′UTR flanked with 30 +/− bp. For MUT oligonucleotides, point mutations in the respective miRNA binding sites were introduced to diminish the putative interaction between the miRNA and the 3′UTR region (Table S2). Vectors were purified using PhasePrep BAC DNA Kit (Sigma-Aldrich, St. Louis, MO, USA) and verified by Sanger sequencing.

#### 2.5.2. Luciferase Reporter Assay

Validation of the interactions between selected genes and miRNAs was performed in the HEK 293T cells. At least two independent transfections, in three technical repetitions were performed with jetPRIME DNA/siRNA (Polyplus-transfection SA, Illkirch, France) reagents as follows:

- In total, 500 ng of vector containing the 3′UTR WT sequence + 50 µM of the analyzed miRNA mimic (mirVana^®^ miRNA mimic, Invitrogen, Waltham, MA, USA);
- In total, 500 ng of vector containing the 3′UTR WT sequence + 50 µM of the mimic negative control (mirVana™ miRNA Mimic, Negative Control #1, Invitrogen, Waltham, MA, USA);
- In total, 500 ng of vector containing the 3′UTR MUT sequence + 50 µM of the analyzed miRNA mimic (mirVana^®^ miRNA mimic, Invitrogen, Waltham, MA, USA);
- In total, 500 ng of vector containing the 3′UTR MUT sequence + 50 µM of the mimic negative control (mirVana™ miRNA Mimic, Negative Control #1, Invitrogen, Waltham, MA, USA).

Cell lysis was performed with Dual-Glo Luciferase Assay System, (Promega, Madison, WI, USA), 24 h after transfection. The bioluminescence signal of firefly luciferase was measured using the GloMax^®^ 96 Microplate Luminometer (Promega, Madison, WI, USA) in reference to Renilla luciferase.

### 2.6. MiRNA Overexpression

The insert containing the miR-23a + mir-27a cluster was prepared by PCR amplification. Mature sequences of these miRNAs are encoded only 69 bp apart and were therefore amplified together. Primers specific for the genomic sequence harboring the pre-miRNA hairpins, including approximately 100–250 nt flanking sequence on each site, were designed as described previously by Kluiver et al. [13] (Table S1). The PCR products with cohesive ends were cut with restriction enzymes (EcoRI and BamHI), purified and directly cloned into the pCDH-CMV-MCS-EF1α-GreenPuro vector (SBI, Palo Alto, CA, USA). The control empty vector was a kind gift from Dr Monika Drobna-Sledzinska (Institute of Human Genetics, Polish Academy of Sciences, Poznan, Poland). The vectors were packed into lentiviral particles in HEK 293T cells using jetPRIME transfection reagent (Polyplus-transfection SA, Illkirch, France). The lentiviral particles were harvested 48 h after transfection. Three independent transductions were performed with the vector carrying the respective miRNA sequences as well as by the empty vector. The expression of the GFP-puromycin resistance fusion gene from the vector was used for GFP/drug selection of the transduced cells. Efficiency of transduction was analyzed by flow cytometry and the expression construct harboring the respective miRNAs was validated by real-time qPCR with TaqMan^®^ Advanced miRNA Assay prior to Western blot (Wb) analyses.

### 2.7. Western Blots

Total protein was extracted from the transduced cell lines using RIPA buffer (Sigma-Aldrich, St. Louis, MO, USA) with a protease inhibitor cocktail (BioShop, ON, Canada) and the protein concentration was measured using the Bradford assay (Bio-Rad, Hercules, CA, USA). Western blots were conducted according to the standard procedure using Mini Protean system (Bio-Rad, Hercules, CA, USA) as described previously [14]. Anti-TNFAIP3 (A20) (59A426, Thermo Fisher Scientific, Waltham, MA, USA, 1:1000) and anti-GAPDH (ab9485, Abcam, Cambridge, United Kingdom, 1:5000) antibodies were used. Proteins were visualized by the Clarity Western ECL Substrate (Bio-Rad, Hercules, CA, USA). The images were scanned and analyzed using the ChemiDoc XRS+ System (Bio-Rad, Hercules, CA, USA). Relative TNFAIP3 expression was analyzed in reference to GAPDH using the Image Lab software (Bio-Rad, Hercules, CA, USA). The full Western Blots can be found at Figure S2.

## 3. Results

### 3.1. The cHL miRNome and miRNAs Deregulated in cHL

In order to characterize the miRNAome of cHL we performed small RNA sequencing using the HiSeq 4000 Illumina platform (Illumina, San Diego, CA, USA) on seven cHL cell lines. Together, we detected expression of 298 miRNAs in at least three out of the seven analyzed cHL cell lines (Table S3). These miRNAs present the complete cHL miRNAome established in this study, which is, to the best of our knowledge, the most detailed NGS-based miRNA profile of cHL cell lines published so far. The mean expression of the identified miRNAs in the seven cHL cell lines was 3335 CPM (range 8–295 760 CPM). The top five most abundantly expressed miRNA in cHL were hsa-miR-21-5p and hsa-miR-155-5p, both with proven oncogenic roles in cHL, but also members of the let-7 family hsa-let-7f-5p and hsa-let-7a-5p and hsa-miR-92a-3p. The mean expression of the top five miRNA was 128 645 CPM, which is >30 times higher than the average expression of the identified miRNAs.

**Figure 1:**
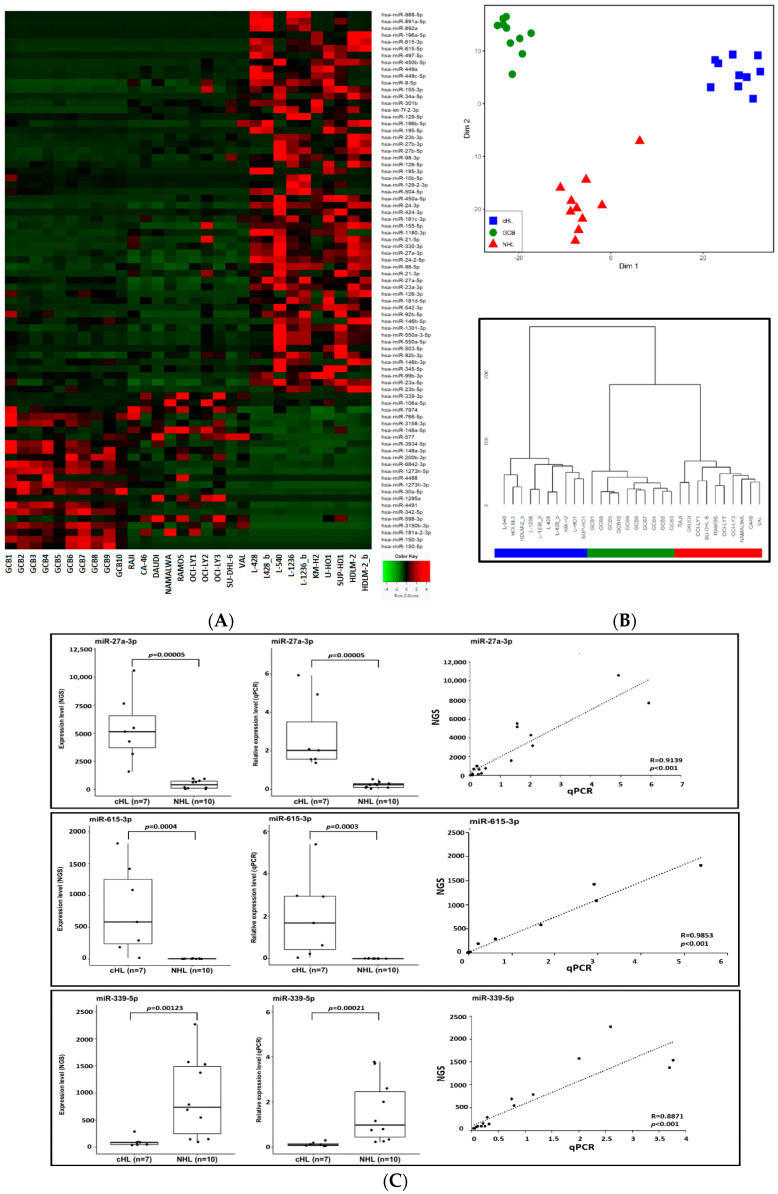
(**A**) Expression heat-map of the 79 miRNAs deregulated exclusively in cHL. GCB—germinal center B-cell pools (*n* = 10) are followed by non-Hodgkin lymphoma cell lines (*n* = 10) and cHL cell lines (*n* = 7) (_b after the cell line name indicates an experimental replication with modified library preparation for NGS. See the Methods section for details). (**B**) Multi-dimensional scaling (MDS) (input matrix was obtained using Canberra distance measure) of cHL, GCB and NHL samples based on expression of the 79 miRNAs deregulated exclusively in cHL (upper panel). Hierarchical clustering of the studied cohorts using Ward’s method with the Canberra distance (lower panel). (**C**) Validation of NGS profiling. Expression of hsa-miR-615-3p, hsa-miR-27a-3p and hsa-miR-339-5p in cHL and NHL cell lines based on NGS and qPCR.

As our main goal was to identify miRNAs deregulated exclusively in cHL we then compared the cHL profiles with profiles of controls that included 10 NHL samples and 10 GCB samples analyzed using the same platform and parameters. By this approach, we found 56 miRNAs to be overexpressed and 23 downregulated in cHL, compared against both control groups (Figure 1A,B). This group of 79 miRNAs contained putative oncomiRs and tumor suppressor miRNAs that may be involved in the pathogenesis of cHL and may contribute to the unique phenotype of HRS cells.

To further validate the NGS-based profiles, we used real-time qPCR TaqMan^®^ Advanced miRNA Assyas (Thermo Fisher Scientific, Waltham, MA, USA) for selected upregulated and downregulated miRNAs as a proof of principle analysis. Three deregulated miRNAs, namely miR-615-3p (upregulated in cHL), miR-27a-3p (upregulated in cHL) and miR-339-5p (downregulated w cHL) were analyzed (Figure 1C). Moreover, the expression of two miRNAs downregulated in cHL, namely miR-148a-3p and miR-148a-5p was validated in our previous manuscript [15]. The real-time qPCR-based expression results were highly convergent with those obtained by NGS, demonstrating the credibility of the small RNA sequencing data.

### 3.2. Biological Processes Deregulated by miRNAs in cHL

We next focused our efforts on the identification of biological processes enriched in the group of genes targeted by the 79 miRNAs deregulated in cHL. In silico analysis using the TargetScan online tool (PMID: **26267216**) resulted in the identification of 1298 genes as potential targets for the overexpressed miRNA in cHL, and 1649 genes as targets for the downregulated miRNAs.

These groups were then analyzed for functional enrichments independently using the PANTHER, STRING and DAVID databases. As GO analyses tend to generate large numbers of enriched biological terms, we compared only the top three biological processes identified for the groups of genes using each of the online tools. By this filtering, we identified the “DNA-binding transcription factor activity” (GO:0003700) (*p* < 0.03) biological process to be significantly enriched using all three databases for the upregulated miRNAs. Hence, this biological process is putatively attenuated in cHL, as a consequence of the activity of the 56 miRNAs. This corresponds well with the known characteristic of HRS cells, which silence B-cell specific transcription factor activity. There was no enriched GO term found in all three databases for the group of downregulated miRNAs; however, one process, the “transcription regulatory region sequence-specific DNA binding” (GO:0000976), was returned by two out of three tools. Therefore, these putatively upregulated genes also seem to be involved to some extent in the regulation of transcription (Table 1).

In light of these results, we further focused on deregulated miRNAs and their putative interactions with B-cell specific transcription factors SPI1 and ELF-1 attenuated in cHL [5,16] and also the NF-ĸB inhibitor TNFAIP3 recurrently inactivated in this lymphoma [17]. We selected the putative hsa-miR-27a-5p:SPI1, hsa-miR-330-3p:ELF-1 (two miRNA binding sites were tested), hsa-miR-542-3p:ELF1, hsa-miR-450b-5p:ELF-1 and the hsa-miR-23a-3p:TNFAIP3 interactions for experimental testing (Table 2).

### 3.3. Functional Validation of miRNA-mRNA Interactions for Selected Candidates

**Figure 2:**
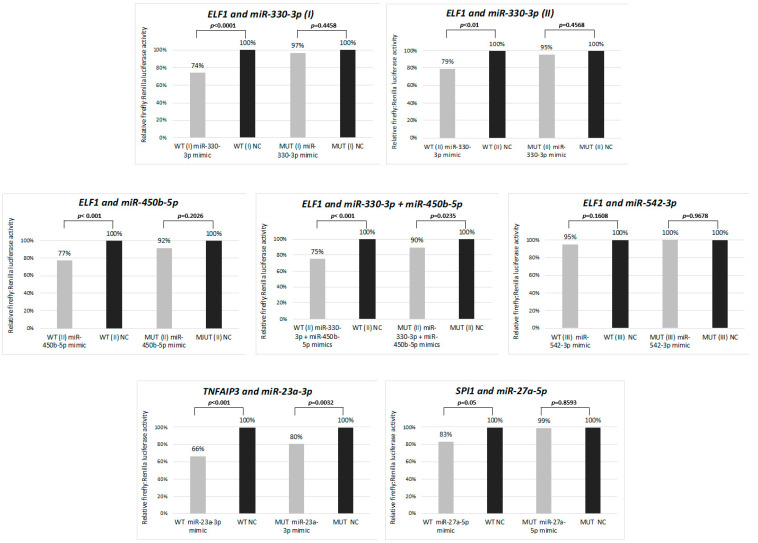
Interaction between selected miRNAs and their target genes (*ELF1, TNFAIP3* and *SPI1*) validated by dual-luciferase reporter assay in HEK 293T cell line. WT: wild type 3′UTR, MUT: mutant 3′UTR, with point mutations in miRNA binding site. Mean from two independent experiments performed in triplicate is shown.

We performed luciferase reporter assays to analyze whether the selected miRNAs indeed interact with the 3′UTR regions of the genes attenuated in cHL. The strongest reduction (34%, *p* < 0.001) of the luciferase intensity was observed as a consequence of the interaction of the hsa-miR-23a-3p mimic with its respective binding site in the 3′ UTR region of the *TNFAIP3* gene. However, a significant but less intensive signal reduction (20%, *p* = 0.0032) was observed also for the mutant 3′UTR binding site, suggesting that the introduced mutations did not abolish the interaction entirely. Moreover, significant reduction of the luciferase intensity was observed for the hsa-miR-330-3p mimic and *ELF1* (by 26%, *p* < 0.001 for the first and by 21%, *p* < 0.01 for the second binding site) and the hsa-miR-450b-5p mimic and *ELF1* (by 23%, *p* < 0.001). Interestingly, a co-transduction with the two *ELF1* interacting mimics did not have a synergistic effect and resulted in the reduction of signal intensity by 25% (*p* < 0.001). However, for hsa-miR-27a-5p:SPI1, we observed a less intensive reduction of the signal intensity (by 17%, *p* = 0.05) and no significant interaction for hsa-miR-542-3p and *ELF1* (Figure 2). Hence, these results suggest that hsa-miR-330-3p, hsa-miR-450b-5p and hsa-miR-27a-5p, but not hsa-miR-542-3p, negatively regulate the respective transcription factors and that hsa-miR-23a-3p may contribute to constant activation of NF-ĸB in cHL.

**Figure 3:**
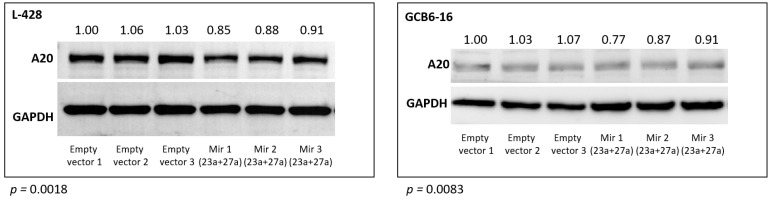
Relative TNFAIP3 (A20) protein abundance after stable overexpression of hsa-mir-23a (23a) and hsa-mir-27a (27a) in L-428 and GCB6-16 cell lines, compared to cells transduced with the empty vector (three independent biological replications are shown).

These findings were further confirmed for the A20 (TNFAIP3) protein using Western blot. The abundance of the A20 protein was significantly reduced in the L428 (*p* = 0.0018) and the GCB6-16 (*p* = 0.0083) cell lines after transduction with the mir-23a + mir-27a expression construct, as compared to the empty vector transduced cell lines (Figure 3).

### 3.4. Validation of miRNA Expression in Microdissected Primary HRS Cells

**Figure 4:**
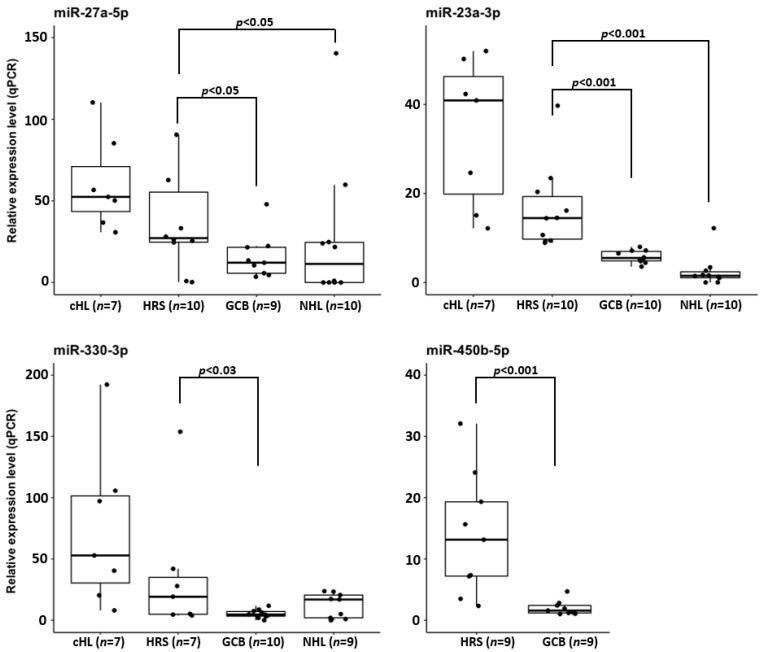
Overexpression of hsa-miR-27a-3p, hsa-miR-23a-3p and hsa-miR-330-3p in cHL cell lines and microdissected HRS cells from cHL cases in comparison to NHL cell lines, and CD77^+^ GCB cell pools and hsa-miR-450b-5p overexpression in microdissected HRS cells in comparison to CD77^+^ GCB cell pools.

To confirm that the overexpression of hsa-miR-23a-3p, hsa-miR-330-3p, hsa-miR-450b-5p and hsa-miR-27a-5p observed in cHL cell lines is not a consequence of cell culturing, we used real-time qPCR molecular probes to analyze the expression of these miRNAs in microdissected HRS cell pools obtained from tumor samples (*n* = 10). The miRNA expression levels observed in HRS cells were compared with the expression in sorted GCB cell pools (*n* = 10) and NHL cell lines (*n* = 10). In line with the findings in cHL cell lines, in the microdissected HRS cells, hsa-miR-23a-3p and hsa-miR-27a-5p were significantly upregulated (*p* < 0.001 and *p* < 0.05 respectively), compared to both control groups. Similarly, hsa-miR-330-3p expression in the HRS cells was significantly upregulated (*p* < 0.03) versus GCB cells and elevated, albeit not significantly, versus the NHL cell lines (*p* = 0.126). Moreover, hsa-miR-450b-5p was significantly upregulated in the HRS cells (*p* < 0.001) compared to in the GCB pools (cHL and NHL cell lines were not included in miR-450b-5p expression analysis) (Figure 4).

Thus, primary HRS, similarly to cHL cell lines, overexpress these four miRNAs. Moreover, these data indicate that hsa-miR-27a-5p and hsa-miR-23a-3p overexpression might be a unique feature of cHL miRNome.

## 4. Discussion

In this study, we established a complete profile of 298 miRNAs expressed in seven commonly used cHL cell lines. Out of these, 126 miRNAs showed a mean expression level of at least 100 CPM, therefore these miRNAs probably exert biological effects in the cells. More specifically, we identified a group of 43 strongly expressed miRNAs (CPM > 1000 and <10,000) and a further 14 miRNAs with very high expression (CPM > 10,000) topped by hsa-let-7f-5p, hsa-miR-155-5p (CPM > 100,000) and especially hsa-miR-21-5p (CPM~300,000) being the most abundantly expressed miRNA in the studied cHL cell lines. The two most abundantly expressed miRNAs in cHL, namely hsa-miR-21-5p and hsa-miR-155-5p, are particularly important in cHL pathogenesis, and were reported to be overexpressed in cHL in earlier studies [18,19,20,21,22,23]. Hsa-miR-21-5p was shown to act via targeting *BTG2* and *PELI1* mRNAs protecting the HRS cells from apoptosis [24] and hsa-miR-155-5p via targeting the tumor suppressor *NIAM* known to promote lymphomagenesis upon silencing [18,25,26]. Importantly, hsa-miR-155-5p contributes also to the constitutive activation of NFĸB in HRS cells, as it was shown to interact with the inhibitor of NFκB kinase subunit epsilon—*IKBKE* [21]. In addition, hsa-let-7f-5p was reported previously in cHL. Its exact function remains unknown; however, other studies suggested its role in silencing pro-apoptotic genes [27].

To systematically identify miRNAs deregulated exclusively in cHL cell lines, we compared their expression with NHL cell lines, as well as sorted GCB cells. This revealed 79 differentially expressed miRNAs in cHL compared to both control groups (56 upregulated and 23 downregulated). The expression profile of deregulated miRNAs in cHL established in our study shows only an average overlap with the profile reported by Yuan et al., who used a similar experimental approach based on small RNA-seq of cHL cell lines (*n* = 4) [28]. The authors reported overexpression of 55 miRNAs in cHL cell lines, out of which 25/55 (45%) overlap with our study, and, similarly, the authors report 29 downregulated miRNAs out of which 9/29 (31%) where found also in our study. However, it should be noted that we aimed to identify miRNAs deregulated exclusively in cHL therefore the expression of the candidate miRNAs was compared not only versus GCB cells but also other lymphomas which likely explains the relatively high discrepancy. Similarly, there is only minimal overlap with earlier reports on miRNA expression in primary microdissected HRS cells. For example, Van Vlierberghe reported the overexpression of 30 miRNAs in primary HRS cells using a miRNA RT kit, out of which five (17%) were overexpressed, and none of the downregulated ones were identified in our analysis [20].

Despite the low overlap between the discussed studies, there emerges a common group of miRNAs found overexpressed in all three analyses in the cHL cell lines and primary HRS cells (for details see Figure S1). These include hsa-miR-9-5p, hsa-miR-24-3p, hsa-miR-196a-5p, hsa-miR-21-5p and hsa-miR-155-5p, although the latter two, with fold change > 3 times in cHL, were not classified by Yuan et al. as overexpressed, probably because of the strict selection criteria applied in the study. Four miRNAs from this list: hsa-miR-9-5p [29,30], hsa-miR-21-5p [24], hsa-miR-24-3p [28] and hsa-miR-155-5p [18,25,26] have a proven oncogenic role in cHL. The role of hsa-miR-196a-5p in the pathogenesis of cHL requires further experimental validation, however it was recently reported that this miRNA promotes cell proliferation, migration and invasion of colorectal cancer. Moreover, what is especially interesting with regard to cHL pathology is that it acts via the inhibition of IκBα, which could potentially contribute to the constant hyperactivity of NFĸB [31].

As the putative target genes of the miRNAs found overexpressed in our study were significantly enriched in functions described as “DNA-binding transcription factor activity” (GO:0003700), we focused on the analysis of miRNAs that could potentially target transcription factors. We speculated that this mechanism likely contributes to the silencing of transcription factors attenuated in cHL that, as a consequence, leads to the loss of B-cell phenotype of HRS cells. In fact, several reports provide evidence that miRNAs are involved in regulation of all stages of mammalian B-cell development [6,32,33]. We confirmed experimentally that hsa-miR-330-3p, hsa-miR-450b-5p, hsa-miR-27a-5p and hsa-miR-23a-3p interact with 3′ UTR sites of *ELF1* (miR-330-3p and miR-450b-5p), *SPI1* (miR-27a-5p) and *TNFAIP3* (miR-23a-3p). *SPI1* (PU.1) and *ELF1* encode transcription factors of known important B-cell development. More specifically, PU.1 has a proven role in the regulation of Igκ transcription and rearrangement, as does ELF1 in the activation of several B-cell specific tyrosine kinases in mice that are important for proper BCR signaling [34,35]. Both factors are recurrently attenuated in cHL [5,16]. However, TNFAIP3 is a ubiquitin-modifying enzyme that targets the RIP1 protein for degradation. RIP1, in turn, is necessary to activate the IKK complex and subsequent NF-κB activation [36]. The importance of the miRNA:mRNA interactions described here is moreover stressed by the fact that these miRNAs were found to be overexpressed, not only in the cHL cell lines, but also in primary microdissected HRS cells.

These newly observed interactions were not previously linked to cHL pathogenesis. However, Guo et al. observed recently, using in vivo mouse and in vitro human cell models, that has-miR-23a-3p regulates inflammation and apoptosis by interacting with *TNFAIP3* in atherosclerosis [37]. In line, this was the strongest interaction observed in our reporter assay experiments, suggesting that overexpression of this miRNA is an alternative and most probably a more subtle mechanism to the recurrent loss of function genomic alterations targeting *TNFAIP3* in cHL [17]. We further validated this interaction on the protein level. The induced overexpression of hsa-miR-23a-3p in L428 (a cHL cell line showing genomic amplification of the *TNFAIP3* gene locus and exceptionally strong expression of the protein) and GCB6-16 (immortalized GCB-cell derived cell line) resulted in the decrease of TNFAIP3 protein abundance.

Hsa-miR-23a-3p, together with hsa-miR-27a-5p, is expressed from the same lncRNA transcript (LOC284454) as the cHL oncogenic hsa-miR-24-3p, which is known to be upregulated in cHL cell lines [28]. However, in the GFP assay reported in the cited study, hsa-miR-23a-3p silencing in four tested cHL cell lines did not result in measurable growth retardation of the HRS cells. On one hand, this may suggest that hsa-miR-23a-3p is overexpressed by a passenger effect, together with the oncogenic miRNA encoded by the lncRNA. On the other hand, hsa-miR-23a-3p’s inhibitory effect on *TNFAIP3*, as shown in our study and its manifestation on the cellular level in the tested cHL cell lines, is most probably hampered by various compound heterozygous loss-of-function mutations of *TNFAIP3*, such as W142STOP codon/del in L-1236, frameshift in amino acid 99/del in KM-H2, or frameshift in amino acid 174/del in HDLM-2 [17].

The involvement of hsa-miR-330-3p and hsa-miR-450b-5p in lymphomagenesis has not been reported so far. However, the oncogenic function of these miRNAs was demonstrated in several solid tumors including hsa-miR-330-3p in lung cancer [38] and breast cancer [39] and hsa-miR-450b-5p in colorectal cancer [40].

## 5. Conclusions

In conclusion, we present a detailed miRNome of the seven commonly used cHL cell lines and confirm the leading role of the hsa-miR-9-5p, hsa-miR-21-5p, hsa-miR-24-3p and hsa-miR-155-5p in cHL pathogenesis, as shown in previous studies. Moreover, we demonstrate four miRNAs that we found overexpressed in cHL cell lines, as well as microdissected HRS cells: hsa-miR-330-3p, hsa-miR-450b-5p, hsa-miR-27a-5p and hsa-miR-23a-3p to interact with respective mRNAs of TFs and *TNFAIP3* attenuated in cHL pathogenesis. Together, these findings constitute a potential alternative silencing mechanism of B-cell specific TF and of NF-ĸB inhibitors in HRS cells.

## Figures and Tables

**Table 1:** GO enrichment analysis for cHL deregulated miRNA target genes.

	GO Analysis of Target Genes of miRNAs Upregulated in cHL		
Top 3 GO Terms (*p* < 0.05)	PANTHER Database	STRING Database	DAVID Database
1	DNA-binding transcription factor activity, RNA polymerase II-specific (GO:0000981)	**DNA-binding transcription factor activity (GO:0003700)**	**DNA-binding transcription factor activity (GO:0003700)**
2	**DNA-binding transcription factor activity (GO:0003700)**	molecular function regulator (GO:0098772)	protein binding (GO:0005515)
3	molecular function regulator (GO:0098772)	transcription regulator activity (GO:0140110)	regulation of transcription, DNA-templated (GO:0006355)
	**GO analysis of target genes of miRNAs downregulated in cHL**		
**Top 3 GO terms (*p* < 0.05)**	**PANTHER database**	**STRING database**	**DAVID database**
1	**transcription regulatory region sequence-specific DNA binding (GO:0000976)**	DNA-binding transcription factor activity, RNA polymerase II-specific (GO:0000981)	transcriptional activator activity, RNA polymerase II core promoter proximal region sequence-specific binding (GO:0001077)
2	RNA polymerase II regulatory region DNA binding (GO:0001012)	**transcription regulatory region sequence-specific DNA binding (GO:0000976)**	neuron migration (GO:0001764)
3	transcription regulatory region DNA binding (GO:0044212)	RNA polymerase II regulatory region sequence-specific DNA binding (GO:0000977)	RNA polymerase II core promoter proximal region sequence-specific DNA binding (GO:0000978)

**Table 2:** miRNA:mRNA interactions tested experimentally.

MiRNA in cHL	MiRNA	Target Gene	UTR miRNA Binding Site [Based on TargetScan Database]			Interaction Confirmed in the Luciferase Assay	Gene Function
			Binding Sites Conservation	Number of Binding Sites	Characteristic of Binding Sites		
UPREGULATED	hsa-miR-27a-5p	SPI1 (PU.1)	poorly conserved	1	7mer-m8	YES	B-cell related transcription factor
	hsa-miR-330-3p	ELF-1	poorly conserved	2	7mer-m8, 7mer-m8	YES	B-cell related transcription factor
	hsa-miR-542-3p	ELF-1	poorly conserved	2	7mer-A1, 7mer-m8	NO	B-cell related transcription factor
	hsa-miR-450b-5p	ELF-1	poorly conserved	1	7mer-A1	YES	B-cell related transcription factor
	hsa-miR-23a-3p	TNFAIP3	conserved	1	8mer	YES	negative regulator of NF-kappaB

## Data Availability

The data presented in this study are available on request from the corresponding author.

## Supplementary Materials

The following are available online at https://www.mdpi.com/article/10.3390/cancers13133131/s1, Figure S1: The overlap of deregulated miRNAs in cHL cell lines and primary HRS cells compared to NHL cell lines and GCB cells in previously published studies and this study. Columns represent miRNA expression profiles from the particular studies. Red and green colors indicate overexpressed and downregulated miRNAs, respectively, black color represents no significant change in miRNA expression in the respective study, Figure S2: Uncropped Figure 3, Table S1: Primer sequences used in the study; restriction sites are in lowercase, Table S2: Oligonucleotide sequences used in luciferase assays. MiRNA binding sites in 3′UTRs are underlined; introduced point mutations are in bold; restriction sites are marked in lowercase, Table S3: List of miRNAs expressed in cHL cell lines (miRNome).
