# Supplementary material for: Deregulated miRNAs Contribute to Silencing of B-Cell Specific Transcription Factors and Activation of NF-κB in Classical Hodgkin Lymphoma
Source: Cancers (Basel). 2021 Jun 23;13(13):3131. doi: 10.3390/cancers13133131 (PMC8269295; doi:10.3390/cancers13133131)
Supplement: Supplementary file 1 [file cancers-13-03131-s001.zip › cancers-1241681 supplementary figures.pdf]

cancers

MDPI

Sypplementary Materials: Deregulated miRNAs Contribute to Silencing of B-cell Specific Transcription Factors and Activation of NF-κB in Classical Hodgkin Lymphoma

Julia Paczkowska, Joanna Janiszewska, Adam Ustaszewski, Julia Bein, Marcin Skalski, Agnieszka Dzikiewicz-Krawczyk, Natalia Rozwadowska, Martin-Leo Hansmann, Sylvia Hartmann and Maciej Giefing

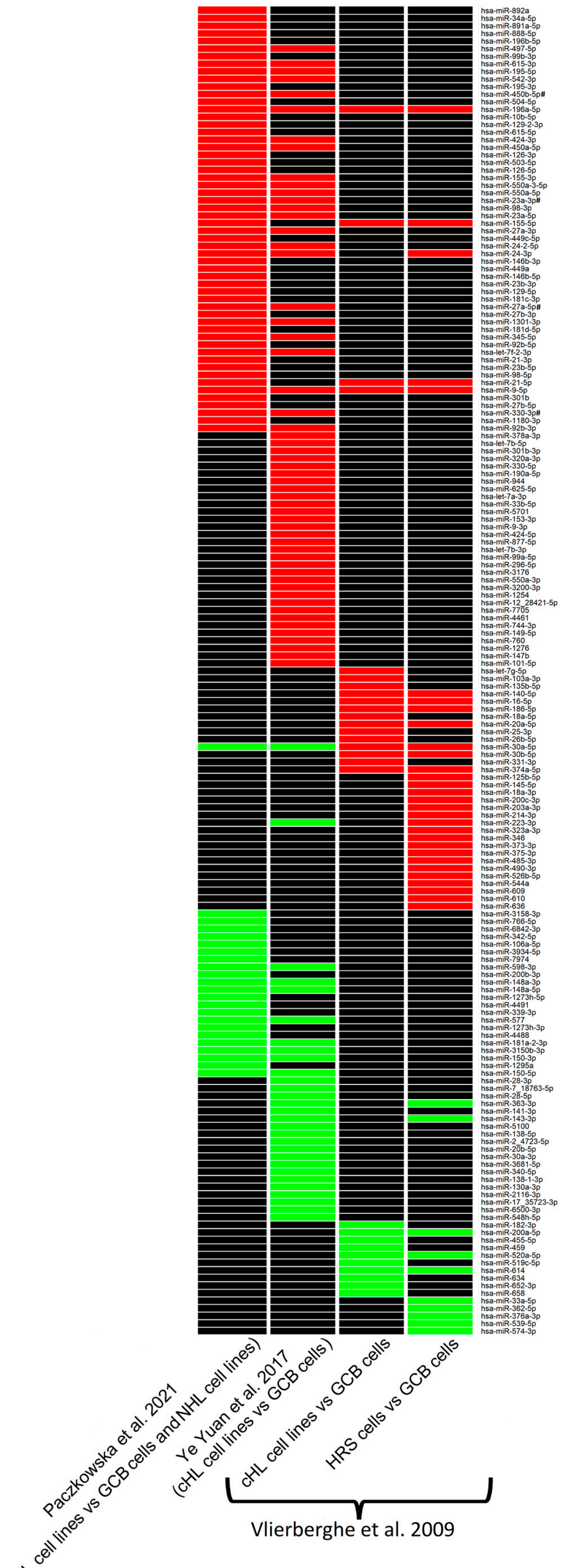
hsa-miR-892a
hsa-miR-34a-5p
hsa-miR-891a-5p
hsa-miR-888-5p
hsa-miR-196b-5p
hsa-miR-497-5p
hsa-miR-99b-3p
hsa-miR-615-3p
hsa-miR-195-5p
hsa-miR-542-3p
hsa-miR-195-3p
hsa-miR-450b-5p#
hsa-miR-504-5p
hsa-miR-196a-5p
hsa-miR-10b-5p
hsa-miR-129-2-3p
hsa-miR-615-5p
hsa-miR-424-3p
hsa-miR-450a-5p
hsa-miR-126-3p
hsa-miR-503-5p
hsa-miR-126-5p
hsa-miR-155-3p
hsa-miR-550a-3-5p
hsa-miR-550a-5p
hsa-miR-23a-3p#
hsa-miR-98-3p
hsa-miR-23a-5p
hsa-miR-155-5p
hsa-miR-27a-3p
hsa-miR-449c-5p
hsa-miR-24-2-5p
hsa-miR-24-3p
hsa-miR-146b-3p
hsa-miR-449a
hsa-miR-146b-5p
hsa-miR-23b-3p
hsa-miR-129-5p
hsa-miR-181c-3p
hsa-miR-27a-5p#
hsa-miR-27b-3p
hsa-miR-1301-3p
hsa-miR-181d-5p
hsa-miR-345-5p
hsa-miR-92b-5p
hsa-let-7f-2-3p
hsa-miR-21-3p
hsa-miR-23b-5p
hsa-miR-98-5p
hsa-miR-21-5p
hsa-miR-9-5p
hsa-miR-301b
hsa-miR-27b-5p
hsa-miR-330-3p#
hsa-miR-1180-3p
hsa-miR-92b-3p
hsa-miR-378a-3p
hsa-let-7b-5p
hsa-miR-301b-3p
hsa-miR-320a-3p
hsa-miR-330-5p
hsa-miR-190a-5p
hsa-miR-944
hsa-miR-625-5p
hsa-let-7a-3p
hsa-miR-33b-5p
hsa-miR-5701
hsa-miR-153-3p
hsa-miR-9-3p
hsa-miR-424-5p
hsa-miR-877-5p
hsa-let-7b-3p
hsa-miR-99a-5p
hsa-miR-296-5p
hsa-miR-3176
hsa-miR-550a-3p
hsa-miR-3200-3p
hsa-miR-1254
hsa-miR-12_28421-5p
hsa-miR-7705
hsa-miR-4461
hsa-miR-744-3p
hsa-miR-149-5p
hsa-miR-760
hsa-miR-1276
hsa-miR-147b
hsa-miR-101-5p
hsa-let-7g-5p
hsa-miR-103a-3p
hsa-miR-135b-5p
hsa-miR-140-5p
hsa-miR-16-5p
hsa-miR-186-5p
hsa-miR-18a-5p
hsa-miR-20a-5p
hsa-miR-25-3p
hsa-miR-26b-5p
hsa-miR-30a-5p
hsa-miR-30b-5p
hsa-miR-331-3p
hsa-miR-374a-5p
hsa-miR-125b-5p
hsa-miR-145-5p
hsa-miR-18a-3p
hsa-miR-200c-3p
hsa-miR-203a-3p
hsa-miR-214-3p
hsa-miR-223-3p
hsa-miR-323a-3p
hsa-miR-346
hsa-miR-373-3p
hsa-miR-375-3p
hsa-miR-485-3p
hsa-miR-490-3p
hsa-miR-526b-5p
hsa-miR-544a
hsa-miR-609
hsa-miR-610
hsa-miR-636
hsa-miR-3158-3p
hsa-miR-766-5p
hsa-miR-6842-3p
hsa-miR-342-5p
hsa-miR-106a-5p
hsa-miR-3934-5p
hsa-miR-7974
hsa-miR-598-3p
hsa-miR-200b-3p
hsa-miR-148a-3p
hsa-miR-148a-5p
hsa-miR-1273h-5p
hsa-miR-4491
hsa-miR-339-3p
hsa-miR-577
hsa-miR-1273h-3p
hsa-miR-4488
hsa-miR-181a-2-3p
hsa-miR-3150b-3p
hsa-miR-150-3p
hsa-miR-1295a
hsa-miR-150-5p
hsa-miR-28-3p
hsa-miR-7_18763-5p
hsa-miR-28-5p
hsa-miR-363-3p
hsa-miR-141-3p
hsa-miR-143-3p
hsa-miR-5100
hsa-miR-138-5p
hsa-miR-2_4723-5p
hsa-miR-20b-5p
hsa-miR-30a-3p
hsa-miR-3681-5p
hsa-miR-340-5p
hsa-miR-138-1-3p
hsa-miR-130a-3p
hsa-miR-2116-3p
hsa-miR-17_35723-3p
hsa-miR-6500-3p
hsa-miR-548h-5p
hsa-miR-182-3p
hsa-miR-200a-5p
hsa-miR-455-5p
hsa-miR-459
hsa-miR-520a-5p
hsa-miR-519c-5p
hsa-miR-614
hsa-miR-634
hsa-miR-652-3p
hsa-miR-658
hsa-miR-33a-5p
hsa-miR-362-5p
hsa-miR-376a-3p
hsa-miR-539-5p
hsa-miR-574-3p
Paczkowska et al. 2021
(cHL cell lines vs GCB cells and NHL cell lines)
Ye Yuan et al. 2017
(cHL cell lines vs GCB cells)
cHL cell lines vs GCB cells
HRS cells vs GCB cells
Vlierberghe et al. 2009

Figure S1. The overlap of deregulated miRNAs in cHL cell lines and primary HRS cells compared to NHL cell lines and GCB cells in previously published studies and this study. Columns represent miRNA expression profiles from the particular studies. Red and green colors indicate overexpressed and downregulated miRNAs, respectively, black color represents no significant change in miRNA expression in the respective study.

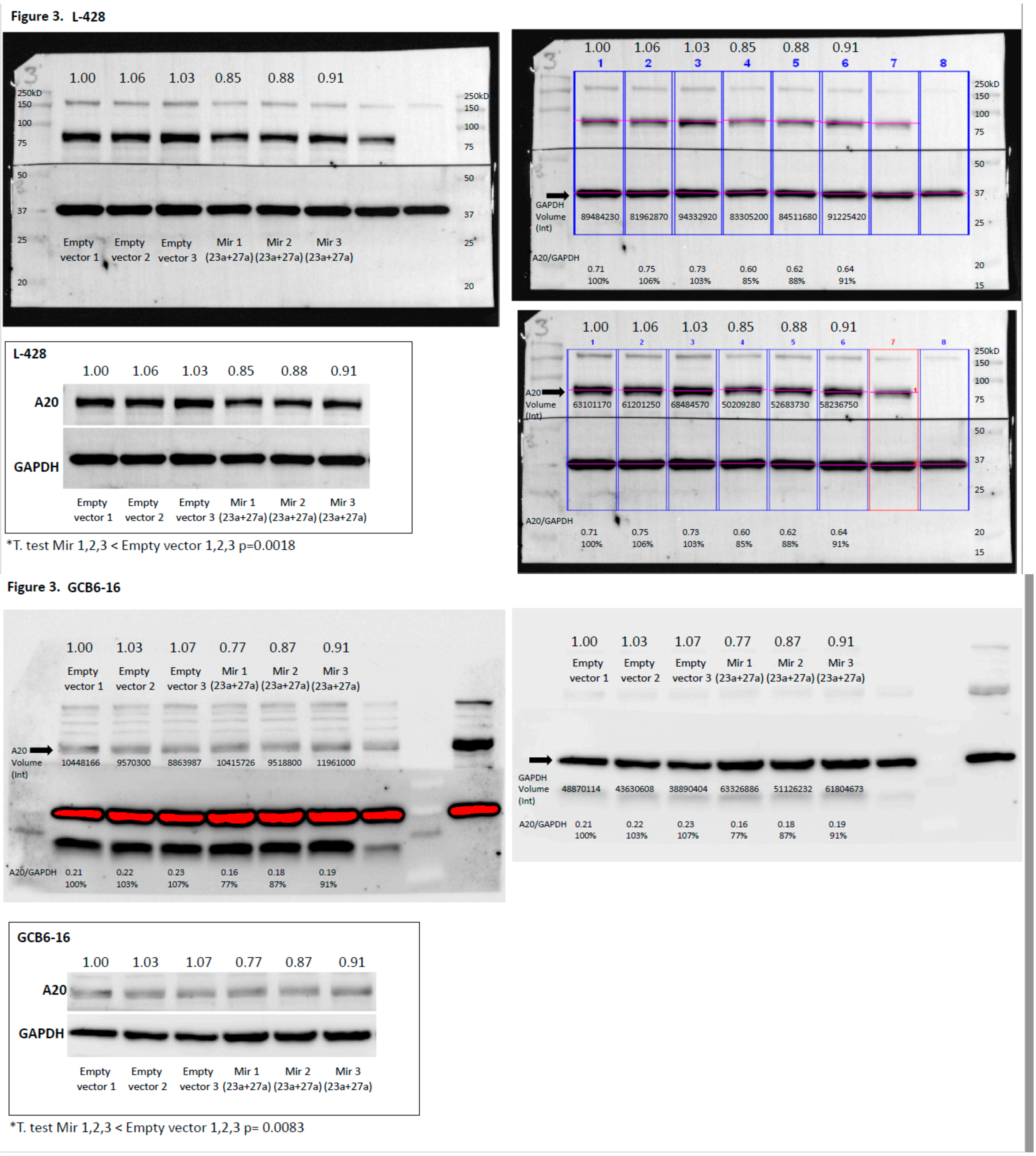

Figure S2. Uncropped Figure 3.
