# Supplementary figures and images for: Deregulated miRNAs Contribute to Silencing of B-Cell Specific Transcription Factors and Activation of NF-κB in Classical Hodgkin Lymphoma
Source: Cancers (Basel). 2021 Jun 23;13(13):3131. doi: 10.3390/cancers13133131 (PMC8269295; doi:10.3390/cancers13133131)

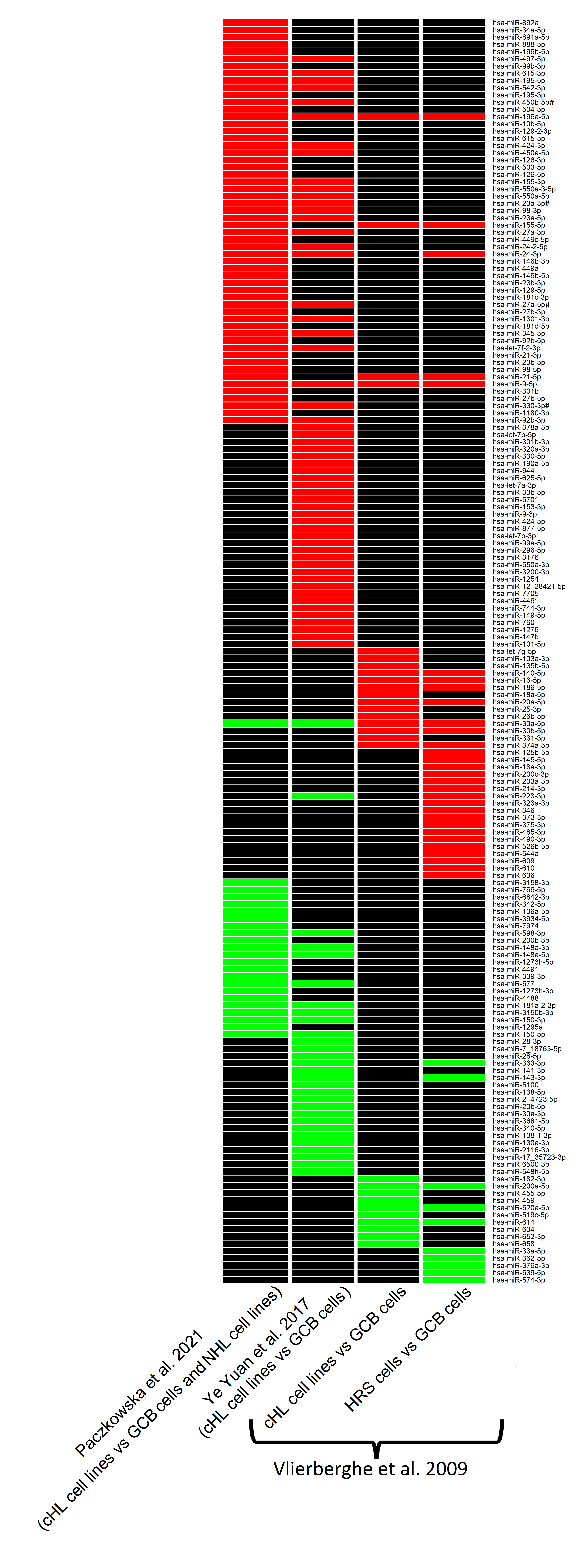

Supplement: Supplementary file 1 [file cancers-13-03131-s001.zip › Figure S1.png]
